# Endoplasmic reticulum-mediated protein quality control in *Arabidopsis*

**DOI:** 10.3389/fpls.2014.00162

**Published:** 2014-04-30

**Authors:** Yidan Liu, Jianming Li

**Affiliations:** Department of Molecular, Cellular, and Developmental Biology, University of MichiganAnn Arbor, MI, USA

**Keywords:** endoplsamic reticulum-associated degradation, *Arabidopsis*, endoplasmic reticulum-mediated quality control, misfolded glycoproteins, receptor-like kinases

## Abstract

A correct three-dimensional structure is crucial for the physiological functions of a protein, yet the folding of proteins to acquire native conformation is a fundamentally error-prone process. Eukaryotic organisms have evolved a highly conserved endoplasmic reticulum-mediated protein quality control (ERQC) mechanism to monitor folding processes of secretory and membrane proteins, allowing export of only correctly folded proteins to their physiological destinations, retaining incompletely/mis-folded ones in the ER for additional folding attempts, marking and removing terminally misfolded ones via a unique multiple-step degradation process known as ER-associated degradation (ERAD). Most of our current knowledge on ERQC and ERAD came from genetic and biochemical investigations in yeast and mammalian cells. Recent studies in the reference plant *Arabidopsis thaliana* uncovered homologous components and similar mechanisms in plants for monitoring protein folding and for retaining, repairing, and removing misfolded proteins. These studies also revealed critical roles of the plant ERQC/ERAD systems in regulating important biochemical/physiological processes, such as abiotic stress tolerance and plant defense. In this review, we discuss our current understanding about the molecular components and biochemical mechanisms of the plant ERQC/ERAD system in comparison to yeast and mammalian systems.

## INTRODUCTION

It is well known that the proper function of a protein strictly depends on its native conformation, but protein folding is a fundamentally error-prone process. The endoplasmic reticulum (ER) is the cellular port of entry for secretory and membrane proteins to enter the secretory pathway and is a folding compartment for proteins to attain their native conformations through interactions with molecular chaperones, sugar-binding lectins, and folding enzymes (Gidalevitz et al., 2013). Misfolded proteins not only lead to functional deficiency but also induce dominant-negative and cellular toxicity effects, and it is thus essential that the ER should possess several highly stringent protein quality control mechanisms to closely monitor the folding process, allowing export of only correctly folded proteins to their final destinations but retaining incompletely/mis-folded proteins for additional rounds of chaperone-assisted folding. A high-efficient ER-mediated protein quality control (ERQC) system can also differentiate terminally misfolded proteins from folding intermediates and/or reparable misfolded proteins, stopping the futile folding cycles of the former proteins and eliminating them via a multistep degradation process widely known as ER-associated degradation (ERAD) that involves ubiquitination, retrotranslocation, and cytosolic proteasome (Smith et al., 2011). Our current understanding of the eukaryotic ERQC/ERAD system derived largely from studies in yeast and mammalian cells. However, recent genetic, biochemical, and cell biological studies in the reference plant *Arabidopsis thaliana* and other model plant species not only identified homologous ERQC/ERAD components but also revealed evolutionarily conserved features as well as unique aspects of the plant ERQC/ERAD mechanisms (Hong and Li, 2012; Huttner and Strasser, 2012; Howell, 2013), especially their connections with the stress tolerance and plant defense pathways.

## *N*-GLYCAN-BASED ER RETENTION MECHANISM

**FIGURE 1:**
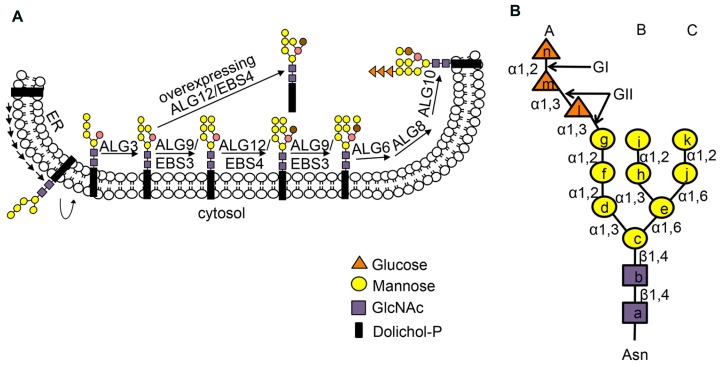
**(A)** Stepwise assembly of *N*-glycan precursor on the ER membrane. The assembly of the *N*-glycan precursor starts at the cytosolic face of the ER membrane by adding two GlcNAc and five Man residues to the membrane-anchored Dol-PP linker. The resulting Dol-PP-Man_5_GlcNAc_2_ flips over into the ER lumen. Four Man residues are sequentially added to the flipped Dol-PP-Man_5_GlcNAc_2_ by three mannosyltransferases, ALG3, ALG9 (known as EBS3 in *Arabidopsis*) and ALG12 (known as EBS4 in *Arabidopsis*) with the ALG9 catalyzing two reactions of adding the terminal α1,2 Man residues on the middle and right branches. Three Glc residues are subsequently added to the right branch, generating the 14-sugar precursor, Dol-PP-Glc_3_Man_9_GlcNAc_2_. Two α1,6 Man residues are marked by brown and salmon color. The Dol-PP linker and different sugar residues are indicated. **(B)** The structure of N-linked Glc_3_Man_9_GlcNac_2_ glycan with three dimannose branches (branch A, B and C). Lower case letters inside sugar residues represent the order of sugar addition. The sugar linkage bonds and enzymes (GI, GII) that remove the three Glc residues are indicated. Figure adapted from Hong et al. (2009).

**FIGURE 2:**
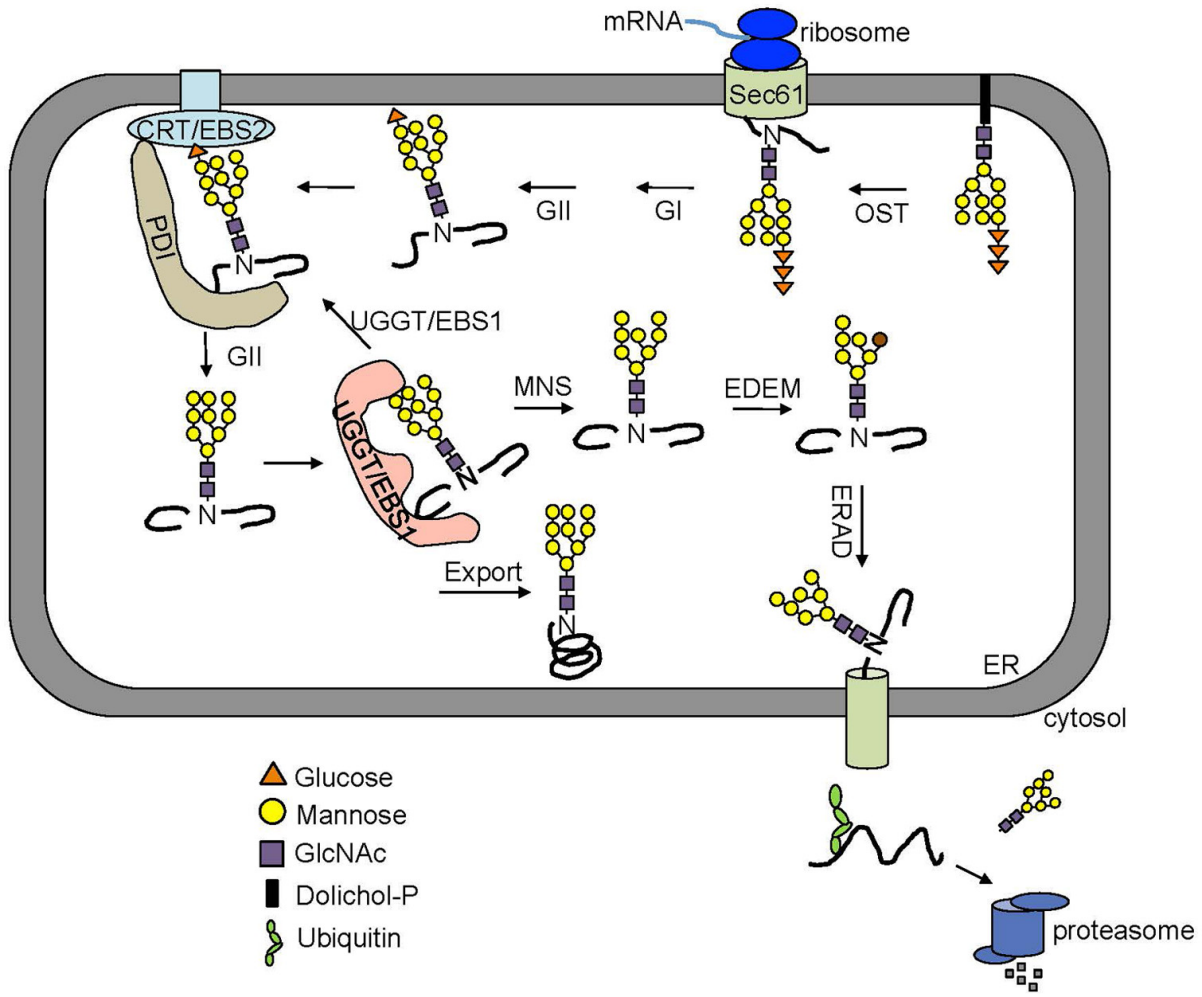
**An overview of the ERQC/ERAD system**. Two Glc residues on the *N*-glycan of a nascent polypeptide are rapidly trimmed by GI and GII right after being transferred from the Dol-PP-linker. The resulting monoglucosylated *N*-glycans bind the two ER lectins CNX and CRT chaperone-assisted folding. The removal of this last Glc by GII releases a mature polypeptide from CNX/CRT. A correctly folded protein can leave the ER while an incompletely/mis-folded glycoprotein is recognized by UGGT (known as EBS1 in *Arabidopsis*) that adds back a Glc residue to the A branch, permitting its reassociation with CNX/CRT. A glycoprotein that fails to gain its native structure within a certain time window is removed from the folding cycle via sequential trimming of the two terminal α1,2 Man residues of the B and C branch by MNS1 (an ER-localized α1,2-Mannosidase, known as MNS3 in *Arabidopsis*) and Htm1/EDEM. A terminally misfolded glycoprotein with α1,6 Man-exposed glycan is selected to enter the ERAD pathway.

Many secretory and membrane proteins are co-translationally glycosylated when entering the ER (Aebi, 2013). The so-called N-linked glycosylation occurs on the asparagine (Asn or N) residues within the Asn-X-Ser/Thr sequons (X indicating any amino acid except proline while Ser/Thr denoting serine/threonine residue) of a nascent polypeptide. This reaction is catalyzed by the enzyme oligosaccharyltransferase (OST), an integral membrane protein complex that transfers a preassembled oligosaccharide precursor Glc_3_Man_9_GlcNac_2_ (Glc, Man and GlcNac denoting glucose, mannose and *N*-acetylglucosamine, respectively) from a membrane-anchored dolichylpyrophosphate (DolPP) carrier to the Asn residue (**Figure 1**; Mohorko et al., 2011). The assembly of Glc_3_Man_9_GlcNac_2_ involves a series of highly specific asparagine-linked glycosylation (ALG) proteins that sequentially add a monosaccharide onto the DolPP linker or a DolPP-linked oligosaccharide precursor (Aebi, 2013; **Figure 1A**). The structure of a N-linked glycan plays an important role in the protein folding and quality control (Aebi et al., 2010). Immediately after transferring of Glc_3_Man_9_GlcNac_2_ to an Asn residue, the terminal and middle Glc residues are removed sequentially by glucosidase I (GI) and glucosidase II (GII), producing a monoglucosylated *N*-glycan, GlcMan_9_GlcNac_2_, which is recognized by the ER chaperone-like lectins, a membrane-anchored calnexin (CNX) and its ER luminal homolog calreticulin (CRT; Caramelo and Parodi, 2008; **Figure 2**). The high-specificity high-affinity binding between GlcMan_9_GlcNac_2_ and CNX/CRT is crucial for folding a nascent polypeptide as CNX/CRT can recruit other ER-chaperones and folding enzymes, including protein disulfide isomerases (PDIs) essential for generating inter/intra-molecular disulfide bonds. The removal of the remaining Glc residue by GII releases the nascent glycoprotein from CNX/CRT, thus terminating its folding process (Caramelo and Parodi, 2008). If the protein folds correctly, it will be transported out of the ER to reach its final destination. However, if the protein fails to attain its native conformation, it will be recognized by UDP-glucose:glycoprotein glucosyltransferase (UGGT), an ER-resident folding sensor consisting of a large non-conserved N-terminal domain presumably involved in recognizing non-native conformations and a smaller highly conserved C-terminal catalytic domain capable of catalyzing a glucosyltransferase reaction using uridyl diphosphate-glucose (UDP-Glc) as a substrate (D’Alessio et al., 2010). As a result, a single Glc is added back to deglucosylated *N*-glycans of the incompletely/mis-folded protein, permitting its reassociation with CNX/CRT and their associated proteins for another round of assisted folding. The alternate reactions of GII and UGGT drive many cycles of dissociation and reassociation of CNX/CRT with an incompletely/mis-folded glycoprotein [widely known as the CNX/CRT cycle (Hammond et al., 1994)], till the protein attains its native conformation (**Figure 2**). It is worthy to mention that the budding yeast (*Saccharomyces* cerevisiae), which is widely used for studying the ERAD process, lacks the CNX/CRT-UGGT system due to the presence of a catalytically inactive UGGT homolog (Meaden et al., 1990).

The *Arabidopsis* genome encodes only one UGGT homolog, and its physiological function was inadvertently found in a study for identifying additional signaling proteins of the plant steroid hormones, brassinosteroids (BR; Jin et al., 2007). A genetic screening for extragenic suppressors of an *Arabidopsis* dwarf mutant *brassinosteroid-insensitive 1-9* (*bri1-9*) led to the discovery of *Arabidopsis* UGGT (also known as EBS1 for EMS mutagenized bri1 suppressor 1; Jin et al., 2007). BRI1 is a cell surface-localized leucine-rich-repeat receptor-like-kinase that function as a BR receptor and contains a single transmembrane domain and 14 putative N-glycosylation sites in its N-terminal extracellular domain (Li and Chory, 1997). The mutant bri1-9, carrying a Ser662-Phe mutation in the BR-binding domain (Noguchi et al., 1999), was found to be retained in the ER by an EBS1/AtUGGT-dependent mechanism and subsequently degraded by a plant ERAD process (Jin et al., 2007; Hong et al., 2009). Loss-of-function mutations in EBS1/AtUGGT compromise such an ER-retention mechanism and allow some bri1-9 proteins to escape from the ER to reach the plasma membrane, resulting in phenotypic suppression of the dwarfism of the *bri1-9* mutant. The same genetic screen also identified CRT3 (Jin et al., 2009), a unique member of the *Arabidopsis* CNX/CRT family consisting of two CNXs and three CRTs, which actually retains bri1-9 via the CRT3-GlcMan_9_GlcNac_2_ binding. Both UGGT and CRT3 were also identified from two other independent genetic screens aiming to identify key regulators of the plant innate immune response to a bacterial translational elongation factor EF-Tu (Li et al., 2009; Saijo et al., 2009). Interestingly, while loss-of-function mutations in AtUGGT/CRT3 led to regaining partial sensitivity to BRs, *atuggt/crt3* mutants were insensitive to elf18, a biologically active epitope of EF-Tu. Further studies showed that both UGGT and CRT3 are absolutely required for the correct folding of EFR (EF-Tu Receptor; Saijo, 2010), a BRI1-like receptor-like kinase that binds elf18/EF-Tu to initiate a plant defense process (Zipfel et al., 2006). The importance of *N*-glycan-mediated folding control was further supported by discoveries that loss-of-function mutations in STT3A, a OST subunit, and GII resulted in significant reduction of the EFR protein abundance, presumably caused by incomplete folding and subsequent degradation (Lu et al., 2009; Haweker et al., 2010; von Numers et al., 2010).

In addition to the glycan-dependent ER retention system, the ER is equipped with additional retention systems to prevent export of misfolded proteins, especially those non-glycosylated ones. One system uses the family of ER-localized HSP70 proteins (known as BiPs), which have a N-terminal ATP-binding domain and a C-terminal substrate-binding domain that recognizes and binds to exposed hydrophobic patches of incompletely/mis-folded proteins in an ATP-dependent manner (Buck et al., 2007). The *Arabidopsis* has three BiP homologs, AtBiP1, AtBiP2 and AtBiP3, all of which were known to exhibit higher levels of gene expression under ER stresses (Sung et al., 2001). In *Arabidopsis*, BiPs were shown to bind both bri1-9 and bri1-5, another mutant variant of BRI1 carrying a Cys69Tyr mutation that destroys a disulfide bridge crucial for the structural integrity of the BR receptor, and were thought to contribute for the ER retention of the two mutant BR receptors (Jin et al., 2007; Hong et al., 2008). BiPs and their associated factors ERdj3B (an *Arabidopsis* ER-localized DNAJ homolog) and SDF2 (the *Arabidopsis* homolog of the murine stromal cell-derived factor 2) are also involved in the biogenesis/folding control of EFR (Nekrasov et al., 2009). BiPs were also known to interact with the orphan heavy chain of a murine IgG1 antibody or an assembly defective form of the trimeric vacuolar storage protein phaseolin in transgenic tobacco plants (Pedrazzini et al., 1997; Nuttall et al., 2002). Another glycan-independent ER retention mechanism relies on mixed disulfide bridges between incompletely/mis-folded proteins with PDIs and related ER-localized oxidoreductases (Reddy et al., 1996; Anelli et al., 2003, 2007). The *Arabidopsis* genome encodes 13 PDI-like proteins (Houston et al., 2005), none of which has been implicated in retaining misfolded proteins. However, a recent study on bri1-5 carrying an orphan cysteine residue (Cys62) suggested involvement of a thiol-mediated retention system in keeping the mutant BR receptor in the ER (Hong et al., 2008). Further biochemical studies are needed to verify this prediction and to identify one or more PDIs that form the predicted mixed disulfide bridge with the orphan Cys62 residue.

## MARKING OF A TERMINALLY MISFOLDED GLYCOPROTEIN FOR ERAD

A protein that fails to attain its native conformation within a given time window is eliminated by ERAD (Vembar and Brodsky, 2008). One of the key events in ERQC is to terminate a futile folding cycle and to deliver an irreparable misfolded protein into the ERAD pathway. Although little is known about the marking mechanism for irreparable non-glycosylated ERAD clients, recent studies indicated that removal of the terminal α1,2-Man residue from the C-branch of *N*-glycan (**Figure 2**), catalyzed by homologous to mannosidase 1 (Htm1) in yeast and ER-degradation enhancing α-mannosidase-like proteins (EDEMs) in mammals, is required for generating the ERAD signal, an exposed α1,6 Man residue on an N-linked glycan (Quan et al., 2008; Clerc et al., 2009; **Figure 1B**). The *Arabidopsis* genome encodes at least two homologs of the yeast Htm1/mammalian EDEMs [known as MNS4 and MNS5 (Liebminger et al., 2009)], but their involvement in a plant ERAD process awaits functional investigation. Nevertheless, recent genetic screening for *Arabidopsis* mutants defective in ERAD of bri1-5/bri1-9 and subsequent molecular cloning and biochemical studies indicated that the glycan ERAD signal is well conserved in plants (Hong et al., 2009, 2012). Loss-of-function mutations in either EBS3 and EBS4 (homologs of the yeast/mammal ALG9 and ALG12, respectively) prevent complete assembly of the *N*-glycan precursor (**Figure 1**), resulting in glycosylation of the two ER-retained mutant BR receptor with truncated *N*-glycan lacking the α1,6-Man residue that would function as the ERAD signal and consequential inhibition of ERAD of bri1-5/bri1-9. In contrast, forcing the addition of the missing α1,6 Man residue to Dol-PP-Man_6_GlcNAc_2_ by overexpression of EBS4/ALG12 in an *Arabidopsis ebs3/alg9 bri1-9* mutant promoted the ERAD of bri1-9 (**Figure 1A**; Hong et al., 2012). Similarly, the ERAD of bri1-9 was presumably accelerated when its N-linked glycans carried a different exposed α1,6 Man residue (the inner α1,6 Man; Hong et al., 2012) caused by a loss-of-function mutation in ALG3 that adds an α1,3 Man to the inner α1,6-Man (Henquet et al., 2008; Kajiura et al., 2010; **Figure 1A**). The exposed inner α1,6 Man residue was shown to function as an alternative ERAD signal in both yeast and mammalian cells (Clerc et al., 2009; Hosokawa et al., 2009).

## RECRUITMENT OF ERAD SUBSTRATES

**FIGURE 3:**
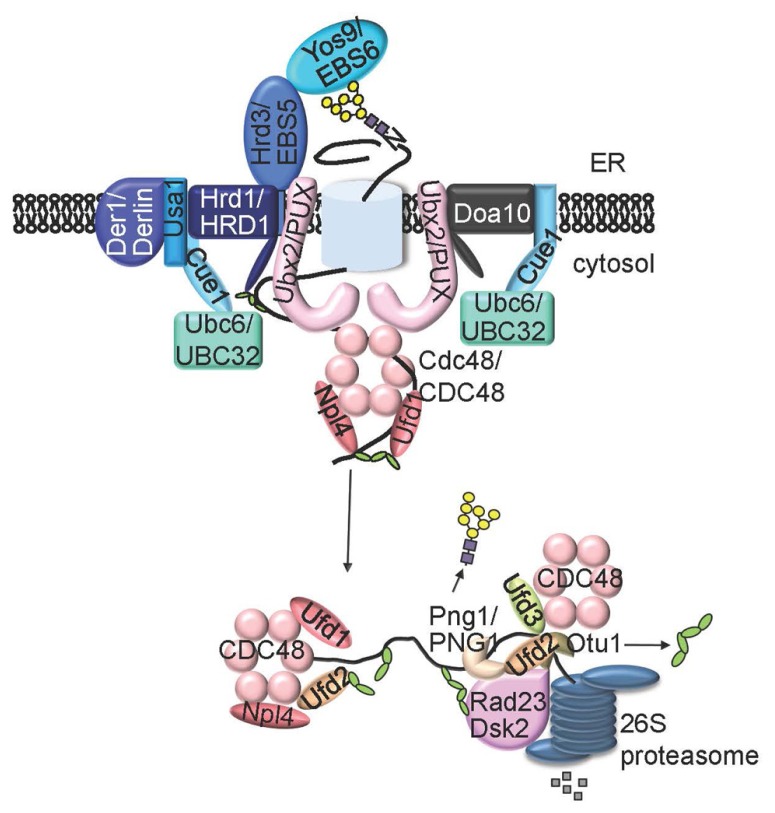
**A model of ERAD system**. An ERAD client that has lesion in membrane-embedded segment (ERAD_M_) or in ER lumen region (ERAD_L_) is recruit by Hrd3 (EBS5 in *Arabidopsis*) and Yos9 (EBS6/OS9 in *Arabidopsis*) to the membrane-anchored E3 ligase Hrd1 (AtHrd1A and AtHrd1B in *Arabidopsis*) complex that also contains Cue1, Ubc6 (UBC32 in *Arabidopsis*), Usa1 and Der1. An ERAD substrate with a folding lesion in the cytosolic domain (ERAD_C_ is recruited to a different ER membrane-anchored E3 ligase complex that contains Doa10 and Ubc6 (or Cue1/Ubc7). The two E3 ligase complexes share similar components on the cytosolic side of the ER membrane, including the substrate-extracting Cdc48-Ufd1-Npl4 trimeric complex and its membrane recruitment factor Ubx2 (one or more AtPUX proteins in *Arabidopsis*). An extracted polyubiquitinated ERAD substrate is processed by Ufd2, Ufd3, Otu1, and/or Png1 (AtPNG1 in *Arabidopsis*), delivered to the cytosolic proteasome with help of Dsk2 and Rad23, and eventually proteolyzed by the 26S proteasome.

The *N*-glycan ERAD signal is decoded by one or two ER luminal lectins, osteosarcoma 9 (OS9, also known as Yos9 in yeast) and XTP3-B (Yoshida and Tanaka, 2010; **Figure 3**). Yos9 and its mammalian homologs contain the mannose-6-phosphate receptor homology (MRH) domain that specifically recognizes and binds *N*-glycans with an exposed α1,6 Man residue (Hosokawa et al., 2010). In addition to OS-9/Yos9, selection of an ERAD client requires another ER resident protein, Hrd3 (HMG-CoA reductase degradation 3) in yeast and Sel1L (Suppressor of lin-12-Like) in mammals (Hirsch et al., 2009), a type I transmembrane protein with a large ER luminal domain consisting of multiple copies of the tetratricopeptide repeat motif. It was believed that Hrd3/Sel1L, exhibiting high affinity binding to exposed hydrophobic amino acid residues on misfolded proteins, makes the initial selection of a potential ERAD client, which is subsequently inspected by OS-9/Yos9 for the presence of an *N*-glycan ERAD signal (Denic et al., 2006; Gauss et al., 2006; **Figure 3**). Such a bipartite ERAD signal of a misfolded domain plus an α1,6-Man-exposed *N*-glycan ensures degradation of only terminally misfolded glycoproteins but not folding intermediates carrying *N*-glycans with no exposed α1,6 Man residue.

The *Arabidopsis* genome has two *Hrd3/Sel1L* homologous genes, *AtSel1A* (also known as *EBS5 or HRD3A* or) and *AtSel1B* (also known as *HRD3B,* an apparent pseudogene) and just one *OS9/Yos9* homolog, *AtOS9* (also known as *EBS6*; Liu et al., 2011; Su et al., 2011, 2012; Huttner et al., 2012). AtSel1A/EBS5 complemented the ERAD-defect of the yeast Δ*hrd3* mutant assayed by ERAD of a mutant variant of carboxypeptidase Y (CPY*; Su et al., 2011), a commonly used ERAD substrate for many ERAD studies in yeast. By contrast, AtOS9 failed to rescue the defective ERAD of CPY* when expressed in a Δ *yos9* yeast strain (Huttner et al., 2012). Interestingly, a chimeric AtOS9-Yos9 protein consisting of the full-length AtOS9 and the Yos9’s C-terminal region (amino acids of 277–542) promoted CPY* degradation in Δ *yos9* yeast cells (Huttner et al., 2012), suggesting that the MRH domain is interchangeable but the Yos9’s C-terminal domain might be crucial for interacting with other components of the yeast ERAD machinery. Loss-of-function mutations in either AtSel1A/EBS5 or AtOS9/EBS6 inhibit ERAD of bri1-5, bri1-9, misfolded EFR (in an *ebs1/uggt* mutant background), and/or the transgenically expressed MLO-1 (Liu et al., 2011; Su et al., 2011, 2012; Huttner et al., 2012), a mutant variant of barley powdery resistance o (MLO) that carries a single amino acid change in the cytoplasmic region and was previously shown to be an ERAD substrate (Muller et al., 2005). As expected, AtSel1A/EBS5 and AtOS9/EBS6 physically interacted with bri1-9 or bri1-5 in a tobacco transient expression system or an *in vitro* pull-down assay (Huttner et al., 2012; Su et al., 2012). Consistent with what was known in yeast and mammalian cells, AtSel1A/EBS5 binds AtOS9/EBS and seems to be required for maintaining the stability of AtOS9/EBS6 (Huttner et al., 2012; Su et al., 2012). These results strongly suggested that the selection mechanism for a terminally misfolded glycoprotein for ERAD is conserved in *Arabidopsis*. It is important to point out that *Arabidopsis* mutants of AtSel1A/EBS6 or AtOS9/EBS6 are hypersensitive to NaCl-induced salt stress, suggesting a relationship between a cellular stress response and an environmental stress pathway (Liu et al., 2011; Huttner et al., 2012). It is quite possible that environmental stresses lead to decreased folding efficiency and increased accumulation of misfolded proteins in the ER, which require a highly efficient ERAD system for their removal to maintain ER homeostasis.

## UBIQUITINATION OF CHOSEN ERAD CLIENTS

Hrd3/Sel1L and Yos9/OS9 not only select irreparable misfolded glycoproteins but also bring the chosen ERAD substrates to the membrane-anchored ERAD complexes responsible for ubiquitination and retrotranslocation. The central component of these ERAD complexes is a polytopic membrane protein with a RING finger-type ubiquitin ligase (E3) activity exposed to the cytosolic surface of the ER membrane, which not only ubiquitinates ERAD substrates but also connects to various ER luminal/cytosolic adapters (Hirsch et al., 2009). Yeast contains at least two distinct E3 ligases, 6 transmembrane-spanning Hrd1 (HMG-CoA reductase degradation) and 14-transmembrane-spanning Doa10 (Degradation of alpha2), that ubiquitinate three different types of ERAD substrates differing in the location of folding lesions: ERAD_L_ (lesion in the ER luminal area), ERAD_M_ (lesion in the transmembrane segment), and ERAD_C_ (lesion in the cytosolic domain; Vashist and Ng, 2004; Carvalho et al., 2006). The Hrd1 complex ubiquitinates ERAD_L__/M_ substrates while the Doa10 complex deals with ERAD_C_ clients. Mammals have at least 9 membrane-bound ERAD E3 ligases (Olzmann et al., 2013), including two Hrd1 homologs (HRD1 and gp78), one Doa10 homolog (TEB4), and several other RING-type E3 ligases such as RING membrane-anchor 1 (RMA1; Younger et al., 2006), whose founding member was initially discovered in *Arabidopsis* (Matsuda and Nakano, 1998).

The *Arabidopsis* genome encodes two Hrd1 homologs (AtHrd1A and AtHrd1B; Su et al., 2011; Huttner and Strasser, 2012), at least two Doa10 homologs (Doa10A/At4g34100 and Doa10B/At4g32670; Liu et al., 2011), and three homologs of RMA1, AtRMA1-AtRMA3 that were shown to be localized to the ER and exhibit *in vitro* E3 ubiquitin ligase activity (Son et al., 2009; **Table 1**), but it remains unclear if plants use distinct E3 ligases to removal different classes of ERAD substrates. Loss-of-function mutations in AtHrd1A or AtHrd1B had no detectable effect on bri1-5/bri1-9 degradation, but simultaneous elimination of the two Hrd1 homologs inhibited degradation of the two mutant BR receptors, indicating that AtHrd1A and AtHrd1B function redundantly in a plant ERAD pathway (Su et al., 2011). By contrast, the role of the two Doa10 homologs in the plant ERAD pathway remains unknown. Two recent genetic studies revealed important regulator roles of Doa10A (also known as SUD1 for SUPPRESSOR OF DRY2 DEFECTS1 or CER9 for ECERIFERUM9) in the cuticle lipid biosynthesis and in controlling the activity but not the protein level of an *Arabidopsis* HMG-CoA reductase (Lu et al., 2012; Doblas et al., 2013). Further studies are needed to determine if the *Arabidopsis* Doa10A is indeed involved in an ERAD pathway that regulates the protein abundance of key regulatory factors or metabolic enzymes involved in the cuticle lipid biosynthesis. Unlike yeast but similar to mammals, plants have additional membrane-anchored RING-type E3 ligases for ERAD. For example, the three *Arabidopsis* RMA1 homologs (Rma1H1) and a hot pepper (*Capsicum annuum*) Rma1H1 are involved in the degradation of a cell surface water channel to regulate its plasma membrane level (Lee et al., 2009). A recent study also suggested that a legume (*Medicago truncatula*) homolog of RMA1 seems to play a role in the regulation of biosynthesis of plant defense compounds, triterpene saponins that share the same biosynthetic precursors with sterols, through regulated degradation of HMG-CoA reductase (Pollier et al., 2013).

**Table 1:** A list of known/predicted components of the *Arabidopsis* ERAD system.

Yeast/human gene name	*Arabidopsis* name	Accession number	Reference
Hrd1/HRD1	HRD1A	At3g16090	Su et al. (2011)
	HRD1B	At1g65040	
Hrd3/SEL1L	EBS5/HRD3A	At1g18260	Su et al. (2011)
	HRD3B	At1g73570	
Yos9/OS-9	EBS6/OS9	At5g35080	Huttner et al. (2012), Su et al. (2012)
Der1/DERLIN	DER1	At4g29330	Kirst et al. (2005)
	DER2.1	At4g04860	Wang et al. (2008), Kamauchi et al. (2005)
	DER2.2	At4g21810	
Ubc6/UBE2J1	UBC32	At3g17000	Kraft et al. (2005), Cui et al. (2012)
	UBC33	At5g50430	
	UBC34	At1g17280	
Htm1/EDEM	MNS4	At5g43710	Liebminger et al. (2009)
	MNS5	At1g27520
Doa10/TEB4	SUD1/CER9/DOA10A	At4g34100	Liu et al. (2011), Doblas et al. (2013), Lu et al. (2012)
	DOA10B	At4g32670	
RMA1	RMA1	At4G03510	Matsuda and Nakano (1998), Son et al. (2009)
	RMA2	At4g28270	
	RMA3	At4g27470	
Ubx2/ERASIN	PUX1	At3g27310	Park et al. (2007), Rancour et al. (2004)
	PUX2	At2g01650	Suzuki et al. (2001), Ueda et al. (2000)
	PUX3	At4g22150	
	PUX4	At4g04210	
	PUX5	At4g15410	
	PUX6	At3g21660	
	PUX7	At1g14570	
	PUX8/SAY1	At4g11740	
	PUX9	At4g00752	
	PUX10	At4g10790	
	PUX11	At2g43210	
	PUX12	At3g23605	
	PUX13	At4g23040	
	PUX14	At4g14250	
	PUX15	At1g59550	
Cdc48/p97	CDC48A	At3g09840	Rancour et al. (2002)
	CDC48B	At2g03670	
	CDC48C	At3g01610	
Npl4/NPL4		At2g47970	
		At3g63000	
Ufd1/UFD1	UFD1	At2g21270	Galvao et al. (2008)
		At4g38930	
		At2g29070	
		At4g15420	
Ufd2/UFD2		At5g15400	Bachmair et al. (2001)
Png1/PNG1	PNG1	At5g49570	Diepold et al. (2007)
Rad23/RAD23	RAD23A	At1g16190	Farmer et al. (2010)
	RAD23B	At1g79650	
	RAD23C	At3g02540	
	RAD23D	At5g38470	

In a typical ubiquitination reaction, ubiquitin is attached to a substrate through a three-step process consisting of activation, conjugation, and ligation catalyzed by an ubiquitin-activating enzyme (E1), ubiquitin-conjugating enzyme (E2), and E3 (Pickart, 2004). In yeast, the Hrd1/Doa10 E3 ligases work together with a membrane-anchored E2 (Ubc6) and two cytosolic E2s (Ubc1 and Ubc7) that are recruited to the ER membrane by an ER anchor protein Cue1 (Hirsch et al., 2009), which also activates both E2 and E3 (Bagola et al., 2013; Metzger et al., 2013). *Arabidopsis* has a total of 37 E2 enzymes (Kraft et al., 2005), including one potential Ubc1 homolog (UBC27), three putative homologs of Ubc7 known to be the cognate E2 for Hrd1 (UBC7 UBC13, and UBC14), and three likely homologs of Ubc6 associated mainly with Doa10 (UBC32, UBC33, and UBC34 each having a predicted transmembrane domain at their C-termini; Liu et al., 2011), but our understanding of the roles of these potential ERAD-participating E2s in the plant ERAD process is extremely limited. One of the *Arabidopsis* Ubc6-like E2 gene, *UBC32*, was recently found to be induced by salt, drought, and ER stress (Cui et al., 2012). Interestingly, the *Arabidopsis ubc32* mutant seedlings are more tolerant whereas *UBC32*-overexpressing transgenic *Arabidopsis* lines are more sensitive to salt and ER stress (Cui et al., 2012). The observed salt tolerance of the *ubc32* mutant is in contrast to the reduced salt tolerance of other known *Arabidopsis* ERAD mutants, including *ebs5/hrd3a*, *ebs6/os9*, and *hrd1ahrd1b* (Liu et al., 2011; Huttner and Strasser, 2012; Huttner et al., 2012). This discrepancy might be explained by different ERAD substrates being degraded by different E3 ligase complexes: ERAD_L/M_ substrates by AtHrd1A/AtHrd1B in association with cytosolic E2s and ERAD_C_ substrates by Doa10 using membrane-anchored E2s. Indeed, UBC32 was found to interact with *Arabidopsis* Doa10B and to stimulate the ubiquitination and degradation of a known ERAD substrate MLO-12, another variant of MLO carrying a single amino acid change in its cytosolic domain (Muller et al., 2005), in a tobacco leaf transient expression experiment (Cui et al., 2012). However, the tobacco result was quite different from the results obtained with the yeast MLO experiment showing that the ERAD of MLO-12 plus two other mutant MLOs (all carrying a cytosolic mutation) used the Ubc7-Hrd1 pathway but was unaffected by either *ubc6* or *doa10* deletion in yeast (Muller et al., 2005). Such inconsistency might be simply caused by heterologous expression of ERAD_C_ substrates in two different eukaryotic systems. Nevertheless, UBC32 was implicated in the Hrd1-mediated degradation of bri1-9 (a presumed ERAD_L_ substrate) as the *ubc32* mutation partially inhibited the degradation of the mutant BR receptor and weakly suppressed the corresponding dwarf phenotype (Cui et al., 2012). The partial inhibition could be attributed to a redundant role of UBC32 with its two close homologs or the potential *Arabidopsis* homologs of Ubc1/Ubc7. However, blast searches failed to find a single homolog of the yeast *Cue1* gene from published sequences of plant genomes and expressed sequence tags (our unpublished results), suggesting that plant ERAD processes might exclusively rely on ER-anchored membrane E2s. Alternatively, plants could recruit cytosolic E2s to the membrane-anchored E3 complexes via yet unknown recruiting factors that share no sequence homology but are functionally similar to Cue1.

The ubiquitination of ERAD substrates, especially those lacking *N*-glycan degradation signals, by the Hrd1 complex requires two additional adapters: U1-Snp1 associating-1 (Usa1; HERP in mammals), an ER membrane protein containing a ubiquitin-like (UBL) motif near it N-terminus and two predicted transmembrane domains in the middle, and Der1 (degradation in the ER; Derlins for Der1-like proteins in mammals), another integral ER membrane protein with four transmembrane segments (Kostova et al., 2007). Usa1 is thought to regulate the stability and/or oligomerization of Hrd1 and to recruit Der1 to the Hrd1 complex (Carvalho et al., 2006, 2010; Horn et al., 2009; Carroll and Hampton, 2010), while Der1 is believed to function either as a receptor for soluble non-glycosylated ERAD substrates or a potential retrotranslocation channel (Lilley and Ploegh, 2004; Ye et al., 2004; Kanehara et al., 2010). The *Arabidopsis* contains no homolog of Usa1/HERP1 but its genome encodes three Der1 homologs whose functional involvement in a plant ERAD pathway awaits detailed genetic and biochemical investigations (Kirst et al., 2005). An earlier study showed that at least two maize Der1 homologs could complement the yeast Δ *der1* mutant, suggesting a potential role for a plant Der1 homolog in an ERAD pathway; however, there is no genetic evidence for proving the hypothesis (Kirst et al., 2005).

## RETROTRANSLOCATION OF ERAD SUBSTRATES

Because the catalytic domains of the ERAD-participating E2s and E3s are on the cytosolic surface of the ER membrane, ERAD substrates need to undergo retrotranslocation for ubiquitination and to access the cytosolic proteasome system for their degradation. However, the molecular nature of this retrotranslocon remains controversial (Hampton and Sommer, 2012). It was previously thought that the Sec61 translocon, which imports nascent polypeptides into the ER lumen during protein biosynthesis, is responsible for retrotranslocation ERAD substrates through the ER membrane (Pilon et al., 1997; Plemper et al., 1997). Other studies suggested that the yeast Der1 and its mammalian orthologs Derlins are the suspected retrotranslocon (Lilley and Ploegh, 2004; Ye et al., 2004). A recent study, however, showed that the E3 ligase Hrd1 itself could serve as the retrotranslocation channel for ERAD_L_ substrates (Carvalho et al., 2010). It is quite possible that all three proteins are capable of retrotranslocation different ERAD substrates involving different adapter proteins.

Compared to the knowledge gained from the yeast and mammalian studies, we know almost nothing about the retrotranslocation step of a plant ERAD pathway. Several earlier studies did suggest the existence of a retrotranslocon in plant cells to move ERAD substrates into the cytosol. A confocal microscopic analysis of subcellular localization of a fusion protein between green fluorescent protein (GFP) with the P-domain of a maize CRT in tobacco leaf protoplasts suggested a retrotransport route from the ER to the cytosol (Brandizzi et al., 2003). In addition, a series of studies revealed that the A chain (known as RTA) of a ribosome-inactivating toxin, ricin that is normally produced as a dimeric protein of RTA covalently linked to a galactose-binding B chain via a single intramolecular disulfide bond and stored in the central vacuole of the endosperm cells of castor bean (*Ricinus communis*), was detected to be deglycosylated and eventual degraded in the cytosol when expressed alone in tobacco leaf protoplasts (Di Cola et al., 2001, 2005; Marshall et al., 2008). It is important to mention that ricin and a few other plant toxins were known to exploit the ERAD pathway to reach their cytosolic targets after being internalized by mammalian cells and retrograde-transported from the cell surface to the ER (Lord et al., 2003). In both yeast and mammalian systems, retrotranslocation of ERAD substrates was driven by ubiquitination (Bagola et al., 2011); however, a recent RTA study using plant protoplasts showed that retrotranslocation is independent of ubiquitination as the lysine-lacking (hence non-ubiquitinated) variant of RTA could still be retrotranslocated from the ER into the cytosol (Di Cola et al., 2005), suggesting that the ubiquitination-retrotranslocation coupling might be substrate-dependent.

## SUBSTRATE EXTRACTION, PROCESSING, AND DELIVERY TO THE PROTEASOME

Without regard to the identity of the actual retrotranslocons, ubiquitinated ERAD clients are extracted from the ER lumen (ERAD_L_ substrates) or ER membrane (ERAD_M/C_ substrates) by a trimeric complex consisting of a homohexameric Cdc48 (p97 or valosin-containing protein in mammals), an AAA-type ATPase and its two substrate-recruiting factors Ufd1 and Npl4 (each having a ubiquitin-binding domain; Wolf and Stolz, 2012). The (CDC48)_6_-Ufd-Npl4 complex itself is recruited to the Hrd1/Doa10 E3 complexes by Ubx2 (VIMP for p97/VCP-interacting membrane protein in mammals), one of the 7 ubiquitin regulatory X (UBX) domain-containing proteins in yeast (13 UBX proteins in mammals; Neuber et al., 2005; Schuberth and Buchberger, 2005, 2008). The current working model posits that extracted ERAD substrates are further processed through antagonistic interactions between an U-box-containing E4 multiubiquitination enzyme Ufd2 and a WD40 repeat-containing protein Ufd3 with unknown enzyme activity plus a deubiquitylating enzyme Otu1, and/or through deglycosylation by the cytoplasmic peptide:*N*-glycanase (PNGase) Png1 (Raasi and Wolf, 2007). The processed ERAD substrates were subsequently delivered to the cytosolic proteasome by Cdc48 in association with two ubiquitin receptors Rad23 and Dsk2, each containing a UBL domain that interacts directly with the cytosolic proteasome and a polyubiquitin-interacting ubiquitin-associated (UBA) domain (Raasi and Wolf, 2007).

The *Arabidopsis* genome encodes three Cdc48 homologs, AtCDC48A, AtCDC48B, and AtCDC48C (Rancour et al., 2002). AtCDC48A was able to complement a yeast *cdc48* mutant (Feiler et al., 1995) and was shown to play a role in the ERAD of a mutant form of MLO and a mutant variant of the *Arabidopsis* vacuolar carboxypeptidase carrying the same Gly-Arg mutation as the yeast CPY* and in the retrotranslocation of RTA and the orphan subunit (RCA A) of another castor bean toxin agglutinin in plant cells (Muller et al., 2005; Marshall et al., 2008; Yamamoto et al., 2010). AtCDC48A is likely to be recruited to the ER membrane by UBX-containing proteins as the *Arabidopsis* genome encodes a total of 15 UBX-containing proteins (known as AtPUXs; **Table 1**), some of which were shown to interact with AtCDC48A (Rancour et al., 2004; Park et al., 2007). It remains to be determined which of the 15 AtPUX proteins are actually involved in recruiting an AtCDC48 to the ER membrane-anchored E3 ligase complexes and play a role in degrading known plant ERAD substrates. Our BLAST searches using the known ERAD components of yeast and mammals as query identified multiple homologs of the Ufd1, Ufd2, Ufd3, Npl4, Rad23, Dsk2 but only a single PNGase homolog in *Arabidopsis* (**Table 1**). The functional involvement of these potential ERAD components in an *Arabidopsis* ERAD process remains unknown except AtPNG1, which was recently shown to contain the suspected PNGase activity and could stimulate the degradation of two mutant variants of RTA in an *N*-glycan-dependent manner in yeast cells (Diepold et al., 2007; Masahara-Negishi et al., 2012).

## CONCLUSION AND CHALLENGES

Despite rapid progress in recent years for identifying molecular components of plant ERQC/ERAD systems and studying their biochemical functions, our understanding of the plant ERQC/ERAD processes remains rather limited, especially about the later stages of the ERAD pathway, such as retrotranslocation, processing of polyubiquitin chains, and delivery (to cytosolic proteasome) of the known plant ERAD substrates. While forward genetic screens in *Arabidopsis* identified the GII-UGGT-mediated CNX/CRT cycle in retaining incomplete/mis-folded glycoproteins and ERAD components that function inside the ER lumen to promote the degradation of the two mutant BR receptors, reverse genetic approaches using T-DNA insertional mutants or RNAi-mediated knockdown of candidate ERAD genes listed in **Table 1** will certainly provide additional knowledge on the plant ERAD mechanisms. Transgenic *Arabidopsis* lines expressing carefully engineered substrates of glycosylated/non-glycosylated ERAD_C_/ERAD_M_ coupled with forward genetic screens and reverse genetic studies will reveal if *Arabidopsis* has several distinct ERAD subpathways using different E3 ligases and adapter proteins that recruits distinct ERAD clients. Similarly, genetic screens for enhancers/suppressors of the *Arabidopsis* wax mutant *cer9* [defective in Doa10A (Lu et al., 2012)] or *drought hypersensitive 2* mutant [that led to independent discovery of Doa10A (Doblas et al., 2013)] could uncover additional ERAD components, reveal unique features of the plant ERAD processes, and a better understanding of the regulatory function of the plant ERAD system in biosynthetic processes. Proteomic studies with the existing *Arabidopsis* mutants of the ERAD E3 ligases could lead to the discovery of additional biochemical pathways and/or physiological processes regulated by the plant ERAD machinery. However, the biggest challenges for the plant ERQC/ERAD research is whether the forward genetic approach in *Arabidopsis* could identify novel ERQC/ERAD components that haven’t been discovered in other eukaryotic systems and if the combination of the *Arabidopsis* genetics with cutting-edge biochemical studies in *Arabidopsis* and transient expression systems could reveal novel biochemical functions of known or predicted ERAD components and provide satisfactory answers to some of the outstanding questions of the general ERQC/ERAD research field.

## Conflict of Interest Statement

The authors declare that the research was conducted in the absence of any commercial or financial relationships that could be construed as a potential conflict of interest.
